# Convolutional Neural Network for the Detection of End-Diastole and End-Systole Frames in Free-Breathing Cardiac Magnetic Resonance Imaging

**DOI:** 10.1155/2017/1640835

**Published:** 2017-07-26

**Authors:** Fan Yang, Yan He, Mubashir Hussain, Hong Xie, Pinggui Lei

**Affiliations:** ^1^School of Biology & Engineering, Guizhou Medical University, Guiyang, Guizhou Province, China; ^2^State Key Laboratory of Bioelectronics, School of Biological Science and Medical Engineering, Southeast University, Nanjing, Jiangsu Province, China; ^3^Department of Medical Imaging, The Affiliated Hospital of Guizhou Medical University, Guiyang, Guizhou Province, China

## Abstract

Free-breathing cardiac magnetic resonance (CMR) imaging has short examination time with high reproducibility. Detection of the end-diastole and the end-systole frames of the free-breathing cardiac magnetic resonance, supplemented by visual identification, is time consuming and laborious. We propose a novel method for automatic identification of both the end-diastole and the end-systole frames, in the free-breathing CMR imaging. The proposed technique utilizes the convolutional neural network to locate the left ventricle and to obtain the end-diastole and the end-systole frames from the respiratory motion signal. The proposed procedure works successfully on our free-breathing CMR data, and the results demonstrate a high degree of accuracy and stability. Convolutional neural network improves the postprocessing efficiency greatly and facilitates the clinical application of the free-breathing CMR imaging.

## 1. Introduction

CMR imaging has become an accurate and reproducible method for left ventricle (LV) function assessment [1]. Free-breathing CMR imaging is an alternative method for the evaluation of LV function [2, 3]. Compared with standard breath-hold steady-state free precession (SSFP) CMR imaging, free-breathing CMR imaging has short acquisition time and prunes away unnecessary breath-hold step. Therefore, free-breathing CMR imaging is beneficial for patients who are unable to hold their breath in clinical examination [4].

**Figure 1:**
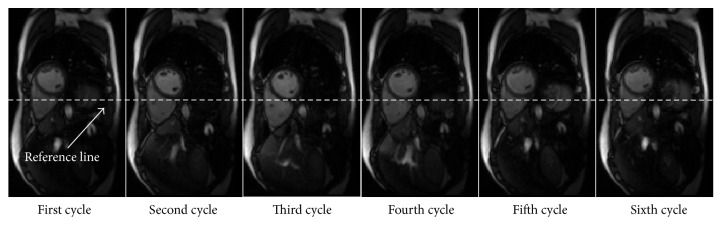
The changing position of LV in ED frame in six cardiac cycles.

The LV function analysis using free-breathing CMR imaging is time consuming. Due to respiratory motion, the LV position is continuously changing along with the diaphragm over multiple cardiac cycles, and the thoracic position of each slice may be mismatched as a result.  Figure 1 illustrates the changing LV position of end-diastole (ED) frames in six cardiac cycles, where the LV position in the third cycle is lower than that in the other cycles. The random choice of one set of cardiac cycle images may lead to an error in LV function evaluation. Therefore, the visual identification for each slice is currently carried out by analyzing ED and end-systole (ES) frames of cardiac cycles at the end-expiration phase [5]. The processing time is about 1~2 min for each slice, where the manual visual identification increases the postprocessing computation, making the procedure extremely laborious. Therefore, an automatic identification method of the ED and ES frames is necessary for improving the free-breathing CMR image processing. Petitjean and Dacher [6] reported that the automatic identification of ED and ES frames is challenging and could be achieved by analyzing the image sequence. Until now, automatic detection of ED and ES frames needed breath-hold, and all frames should be from the same cardiac cycle [7]. Dharanibai and Raina [8] proposed a variance image method for localization of LV. Zhong et al. [9] developed a spectrum-based computer-aided tool to locate the LV. All above-mentioned methods cannot be applied directly for the free-breathing CMR imaging due to the diaphragm motion and can be disturbed by multiple cardiac cycles.

**Figure 2:**
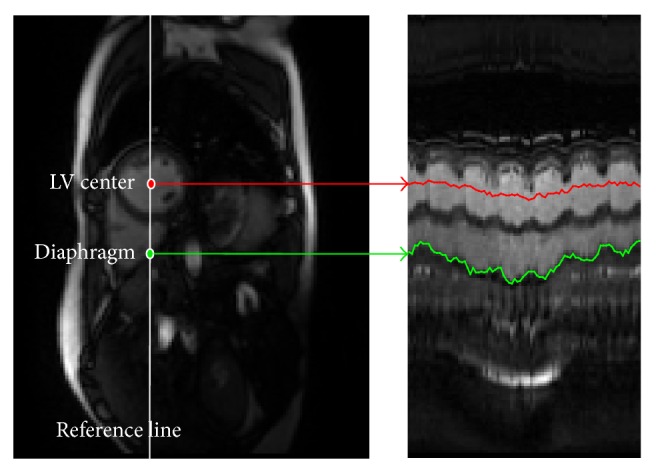
The changing LV center (red) along with the diaphragm (green) over multiple cardiac cycles.

In free-breathing CMR imaging, the ED and ES frames at the end-expiration are essential for LV function analysis. The end-expiration should be determined prior to the detection of the ED and ES frames. In this paper, the LV changing position along with the diaphragm is calculated, and the LV center is adopted to depict the respiratory motion by browsing through all the image sequences.  Figure 2 shows the changing position of the LV center with synchronous diaphragm; the LV center moves across the diaphragm in each frame by putting a reference line on the LV center. With the advent of artificial intelligence, Deep Learning Technology has been developed in medical image processing, such as Convolutional Neural Network (CNN) [10] and Deep Belief Network (DBN) [11]. Avendi et al. [12] introduced CNN to locate the LV position in a deformable-model method and tested it on the MICCAI 2009 LV segmentation challenge database [13]; the results indicated that the method outperforms the other state-of-the art techniques. However, the free-breathing CMR images are different from the MICCAI database. The CNN model needs to be redesigned and fine-tuned for processing these CMR images.

In this paper, we propose a novel method to detect the ED and ES frames of each slice at the end-expiration phase for the free-breathing CMR images. The CNN is designed to locate LV in all frames of each slice. The LV center is located by way of ellipse fitting. The cardiac motion and respiratory motion signals are detected with the changing LV center. With assistance from normalized cross-correlation and blood pool area of LV, the ED and ES frames are determined at the end-expiration stage by respiratory motion signal. The proposed technique is tested on free-breathing CMR images. The experimental results validate its accuracy for detection of the ED and ES frames. The layout of the paper is as follows. The datasets are presented in detail in Section 2. The procedures of the method are presented in Section 3.  Section 4 demonstrates the experimental results.  Section 5 presents discussions and analysis of the results. Finally, the conclusions are given in Section 6.

## 2. Materials

### 2.1. Free-Breathing CMR Data

The study was approved by the Institutional Review Board. Ten healthy subjects (5 males, 5 females; age 25 ± 4; BMI 21.3 ± 1.9), with informed consent, were recruited for the study. The heart function assessments were carried out by using a 3.0 T MR scanner (TIM TRIO, Siemens, Germany). The heart rate was monitored by using ECG. Ten short-axis slices covering the whole heart from apex to base were imaged using a free-breathing 2D real-time SSFP. Karhunen-Loeve transform filter was applied to these slices along the temporal direction to increase the signal-to-noise ratio. The imaging parameters were as follows: slice thickness 8 mm with 2 mm gap, field of view (FOV) = 340 × 287 mm^2^, repetition time/echo time (TR/TE) = 2.5/1.1 ms, matrix size = 160 × 128, TPAT = 4, bandwidth = 1488 Hz/pixel, temporal resolution = 59.5 ms, and cine duration of 5 s for each slice, containing 84 frames covering end-expiration and end-inspiration.

### 2.2. STACOM 2011 LV Segmentation Challenge Database

The data was obtained from the Cardiac Atlas Project Segmentation Challenge [14], which was initiated at the 2011 LV Segmentation Challenge in the 2011 STACOM Workshop. It consisted of 200 subjects with coronary artery disease and myocardial infarction. The dataset is divided into two sets of 100 cases each involving training and validation. As the training set contains the LV contours, we adopt the training set to determine the parameters for localizing the heart region in the CNN model.

## 3. Methods

### 3.1. Heart Region and LV Localization

**Figure 3:**
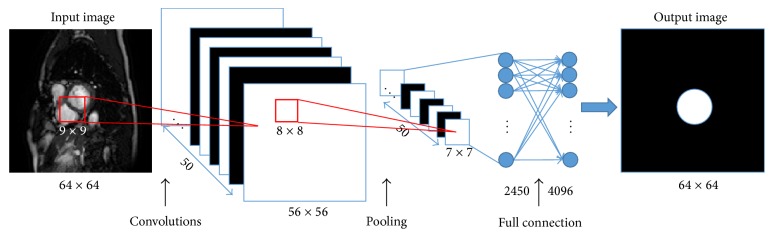
The block diagram of CNN for the heart localization.

Aiming at ED or ES frame identification and LV segmentation, it is necessary to initially locate the LV region in each frame. Previous localization methods need breath-hold imaging and assumed that the heart is the only moving tissue [15, 16]. However, the moving tissue did not only contain the heart, but also the thorax and the diaphragm under free-breathing condition. Our method brings in CNN to locate the LV. The training process is divided into two steps, the heart region localization and the LV localization. In the first step, the original image is padded with zero and downsampled to size 64 × 64 to be the input image. In the convolution layer, 50 convolved maps are captured by adopting 50 filters of size 9 × 9. The weights of the filters are randomly initialized from a Gaussian distribution with fixed zero mean and fixed standard deviation of 0.01. The max pooling of size 8 × 8 and the stride of 8 are taken to downsample the convolved maps to a size 7 × 7. In the end, the max pooling feature layer of size 7 × 7 × 50 is connected to fully connect the layer yielding 4096 outputs. The outputs are reshaped into a mask of size 64 × 64 in which the coordinates of a white circle are taken as the heart region for the input image.  Figure 3 demonstrates the block diagram of CNN for heart localization.

**Figure 4:**
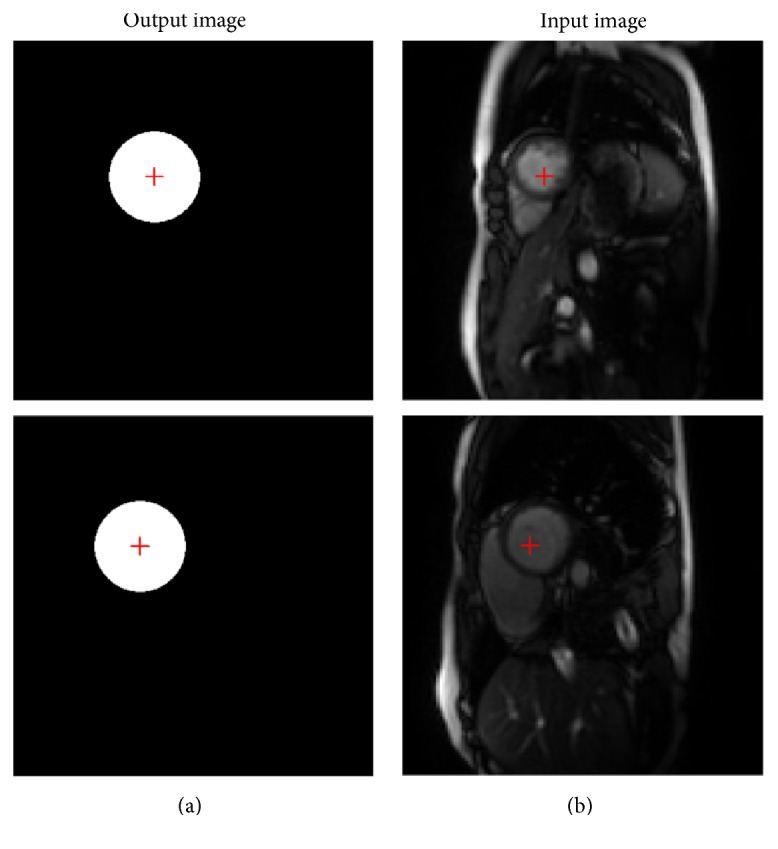
The input images (b) and the corresponding binary masks (a) produced by the trained network. The center of the output image is the same as that of the input image.

In the second step, the free-breathing CMR data is padded with zero and downsampled to size 64 × 64. Then the downsampled data is the input for the trained network produced at the first step. On account of the center coordinates of the white circle in the images generated from the trained network, we discern the same center in the free-breathing CMR images. The output is the corresponding binary mask in the neural network layer.  Figure 4 illustrates the input images and the corresponding output image. The center of the output image is the same as the input image.

**Figure 5:**
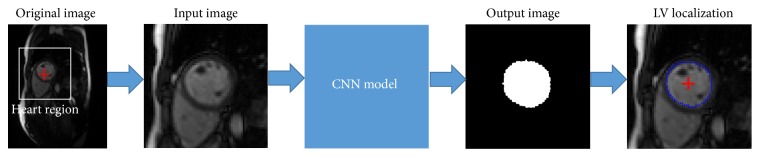
The block diagram of the LV localization.

Afterwards, we choose a heart region of size 64 × 64 whose center is the corresponding center of the input image as demonstrated in Figure 4. Then the heart region of the input image is adopted to train the network for the LV localization. Parameters for the CNN model remain the same as that created in our first step. The input image of size 64 × 64 is considered as the heart region for the original image, and then the output image of size 64 × 64 becomes the binary mask of the LV region. The center of the endocardial contour is determined by the center coordinates of the white region in the output mask.  Figure 5 displays the block diagram for the LV localization according to the LV center and the endocardial contour.

**Figure 6:**
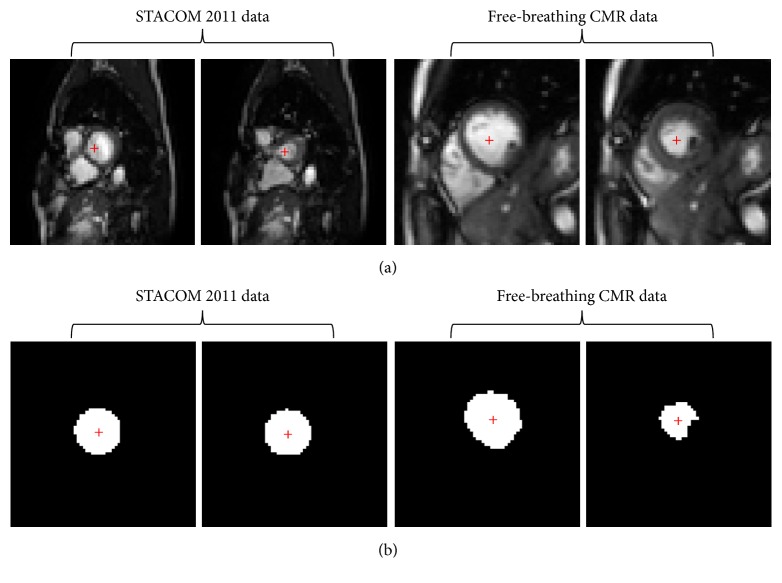
Input image (a) and labeled image (b) of the STACOM 2011 data and free-breathing CMR data.

The network is trained prior to the LV localization. The 100 subjects in the STACOM 2011 database are brought in for parameter training of the heart region localization, where each subject is associated with 170 to 476 images. The remaining 9 subjects of the free-breathing CMR data are trained for the LV localization, where each subject is matched with 840 images. The remaining 1 subject of the free-breathing CMR data is left for effect testing. We augment the training dataset through image translation, rotation, and image intensity alteration. Finally, 1700 to 8400 images are associated with each subject. For the first step, the labeled image of the STACOM 2011 data is created based on the provided contours, where the labeled image is a binary mask having a white round foreground and a black background. In the second step, the labeled image for the free-breathing CMR data is generated by way of region growing method. The seed point is confirmed by manually clicking the LV center on the input image.  Figure 6 demonstrates the input image and the labeled image of the STACOM 2011 data as well as that of the free-breathing CMR data.

### 3.2. The Detection of the LV Center

**Figure 7:**
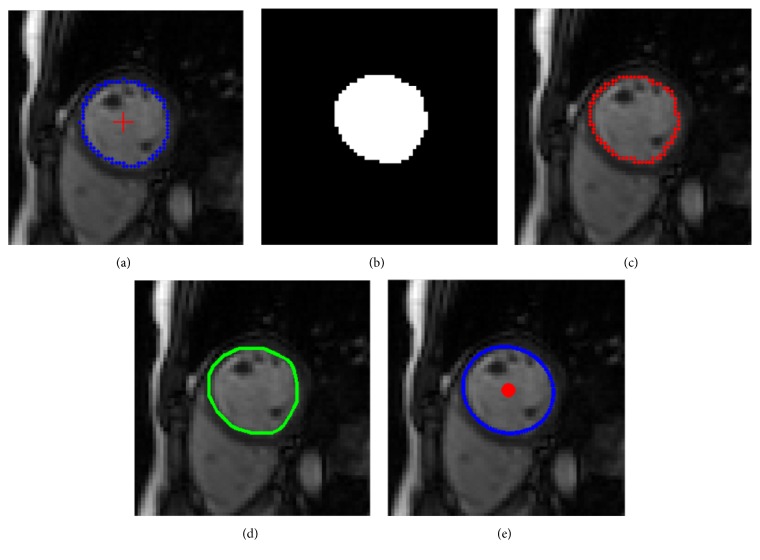
The result of the LV ellipse fitting in one cardiac frame: (a) the center of the endocardial contour; (b) region growing result; (c) blood edge; (d) convex hull curve; (e) LV ellipse fitting curve and the LV center.

Although the LV identification is achieved from the trained network, the center of the segmented LV endocardial contour is inaccurate for detecting the ED and ES frames. The LV center needs to be reconfirmed again. We consider the center of the endocardial contour obtained from CNN model as the seed points for region growing. The optimal segmentation threshold is calculated by the Otsu method [17] for each frame. The neighboring points which are higher than the threshold are added to seed points, and the lower points are denoted as the endocardial contour. Then the LV blood pool is segmented by making use of the output image. In order to decrease the influence of papillary muscles and trabeculations in detecting the LV center, the convex hull of the LV blood pool is implemented before the LV ellipse fitting. Then the least squares method is applied for the LV ellipse fitting and the LV center localization.  Figure 7 presents the LV ellipse fitting curve as well as the convex hull and the LV center.

### 3.3. Respiratory Motion Fitting

**Figure 8:**
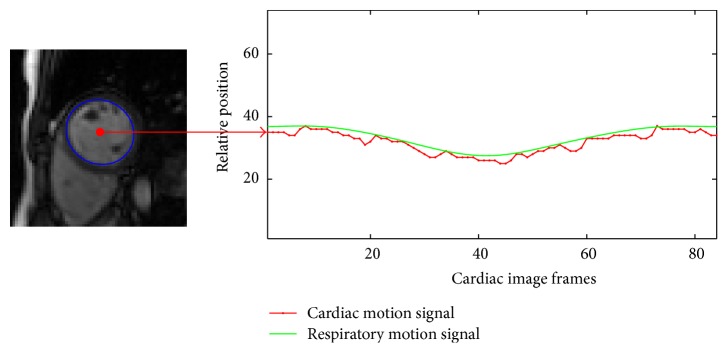
Respiratory motion and cardiac motion signal detection. The horizontal axis represents the cardiac image frames numbered 1 to 84. The vertical axis represents the relative position which is the distance between the LV center and the boundary of the image.

The relative position of the LV center and the boundary of the image are taken as the distance between the LV center and the boundary of the image. Since the heart position is changing along with the diaphragm, the relative position of the LV at the end-expiration is higher than that at the end-inspiration; the center position of the LV is adopted for depicting the respiratory motion. Due to the contraction of LV, the signal curve is unsmooth and exhibits fluctuations. Considering the higher contraction frequency of the heart compared with the respiratory frequency, a low-pass filter is applied for smoothing the curve in order to obtain the respiratory motion signal, and the Hanning window is chosen for weak signal fluctuations.  Figure 8 describes the signal detection of respiratory motion and cardiac motion.

### 3.4. The Detection of the End-Expiration Phase

Free-breathing CMR imaging contains several pairs of ED and ES frames. The ED and ES frames at the end-expiration are dedicated for the LV function analysis. It is critical to identify the end-expiration precisely before locating the ED and ES frames, whereas the maximum and the minimum areas of the LV ellipse fitting correspond to the ED and ES frames separately.

**Figure 9:**
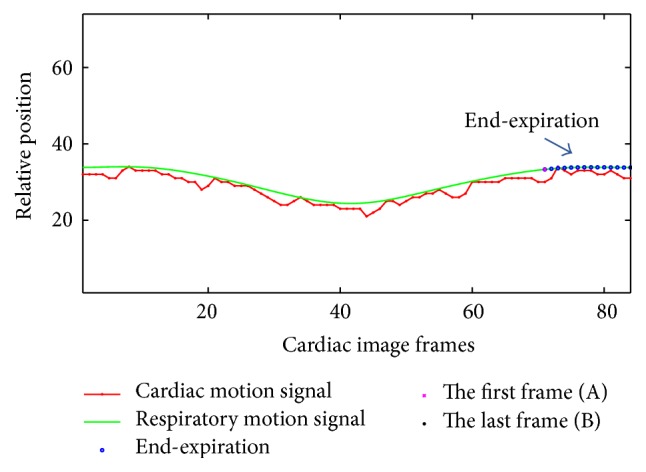
The detection at the end-expiration. The blue circle denotes the end-expiration. Frames (A) and (B) are the first frame and last frame in the end-expiration.

In view of the positions of the ED and ES frames in the image sequence in the same slice, the number of frames (*N*_f_) of one cardiac cycle can be calculated. As the relative position of the end-expiration is higher than that at the end-inspiration, we regard the maximum value of the sum of the relative position of *N*_f_ frames as the end-expiration. Then the *N* cardiac image frames in each slice can be divided into *N* − *N*_f_ + 1 regions and denoted by the following equations:

(1) $$\begin{array}{c} \overset{N_{f}}{\underset{t=1}{\sum }}H\left(\;t\;\right),\overset{N_{f}+1}{\underset{t=2}{\sum }}H\left(\;t\;\right),\ldots ,\overset{N}{\underset{t=N-N_{f}+1}{\sum }}H\left(\;t\;\right), \end{array}$$

where *H*(*t*) stands for the relative position of *t* frames. The maximum sum of *H*(*t*) is considered to be the starting position of the end-expiration. For example, if *N*_f_ is 14 and the total number of frames in each slice is 84, and the maximum value of the sum of *H*(*t*) is 12, then the end-expiration is realized to be covering frames numbers 12 to 25.  Figure 9 exhibits the end-expiration with *N*_f_ frames from the first frame (A) to the last frame (B).

### 3.5. The Identification of the ED and ES Frames

The minimum of the normalized cross-correlation (NCC) is selected to locate the ED and ES frames for the standard breath-hold SSFP cine [7]. The NCC is defined according to the following formula:

(2) NCC=∑m∑nF1m,n−F1¯F2m,n−F2¯∑m∑nF1m,n−F1¯2∑m∑nF2m,n−F2¯2,

where *F*_1_ and *F*_2_ stand for two images and $\bar{F_{1}}$ and $\bar{F_{2}}$ denote the mean value of *F*_1_ and *F*_2_. The NCC value is always in the range [0,1], which denotes the similarity between two images. If the value of NCC is near zero, the two images are regarded as totally different. As the ED and ES frames are different images, the minimum of NCC is used for affirming the ED and ES frames. However, the free-breathing CMR imaging contains several pairs of the ED and ES frames. The NCC cannot be directly applied to the identification of the ED and ES frames. We choose thus a square window of size 40 × 40 whose center is the same as the LV center. Then, we let *w*(*z*, *t*) denote the window of slice *z* at frame *t*, *t* ∈ (*A*, *B*), where *A* and *B* denote the first frame and the frame in the end-expiration, respectively. The value of NCC is computed among all possible image pairs ranging from *w*(*z*, *t*_*A*_) to *w*(*z*, *t*_*B*_) in order to produce the matrix *C*. The index of the minimum of the matrix *C* is denoted as the ED and ES frames separately. Given that the area of the LV ellipse fitting of the ED frame is larger than that of the ES frame, the order of the ED and ES frames is determined by comparing it with the area of the LV ellipse fitting.

## 4. Experiment and Results

**Figure 10:**
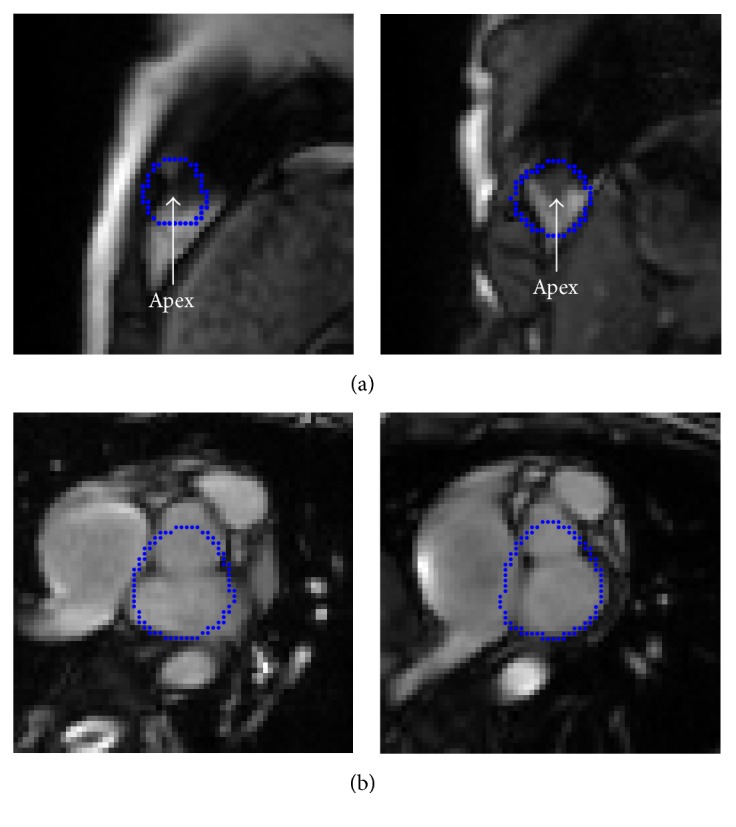
The CMR images of the apical slice (a) and the basal slice (b).

**Figure 11:**
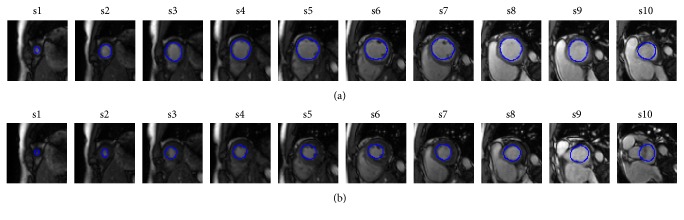
Representative example of the segmented LV endocardial contours of the ED frame (a) and the ES frame (b) by the CNN model from the first slice (apex) to the tenth slice (basal) in the end-expiration.

The network was trained by Caffe in a window operating system as a deep learning framework [18] in which 9 subjects are enrolled for training and 1 subject for testing. The detection method was developed using MATLAB 2014a, which ran on a computer with an Intel 3.4 GHz CPU and 8 GB RAM. Due to dataset shortage, we leave one of the free-breathing CMR datasets for testing each time, and we chose 9 of the free-breathing CMR datasets for training. The training process was repeated 10 times for the second step. For the sake of verifying the detection accuracy, the ED and ES frames of each slice were visually identified by two radiologists and then compared with the automated detection results. The detection results of the ED and ES frames of 10 subjects are illustrated in Tables 1 and 2, respectively. T and F represent the true and false detection results of the ED and ES frames, respectively. S1~s10 denote the ten short-axis slices from apex to base and ED ±* n* or ES ±* n* denote the deviation for ED or ES detection in *n* frames. We consider the ED ±* n* and ES ±* n* as false detection for the detection accuracy. The detection accuracy of the proposed method on 10 subjects is summarized in Table 3. It is shown in Figure 10 that some images in the apical and basal slices lead to detection failure.  Figure 11 presents the segmented LV endocardial contours of the ED frame (a) and the ES frame (b) by the CNN model from the first slice (apex) to the tenth slice (base) in the end-expiration. The average processing time was 0.35 s for each frame for the LV localization. The total detection time of the ED and ES frames was 22 s for each slice.

## 5. Discussion

In this paper, we propose a new method for the ED and ES frames detection in free-breathing CMR imaging covering several cardiac cycles. CNN was adopted to locate the heart region and the LV center. We choose the position of the LV center for fitting the respiratory motion signal. On account of the minimum of the normalized cross-correlation and the area of the LV ellipse fitting in the end-expiration, the ED and ES frames can be identified accordingly. The proposed technique is validated on 10 subjects comprising 8400 images. The average processing time of the LV localization is 0.35 s for each image. The detection time of the ED and ES frames is 22 s for each slice. Compared with the visual inspection method which takes 1 to 2 minutes per image, the proposed method reduces the postprocessing time tremendously.

Table 1 shows the detection results of the ED frame of 10 subjects from the apical slice to the basal slice. The proposed method could detect most ED and ES frames correctly at end-expiration in sequence images for the free-breathing CMR imaging, especially for the mid-ventricular slice. However, as for the apical and basal slices, some ED frames could not be detected successfully. The main reason stems from the small LV chamber or no LV chamber in apical slice, and also certain basal slices were beyond the ventricle and incorporated the left atrium [6]. In the case of other slices, the trained network was able to guarantee the center of the white circle of the output image to be located inside the LV region. Nevertheless, the center point was not the LV center. In order to find the LV center with success, the LV ellipse fitting is adopted and has proven to be the efficient way. The deviation of the ED frame may occur in two or three consecutive frames. Although those consecutive images would have the same area as the LV ellipse fitting and the relative position, the deviation of the ED frame makes it difficult to distinguish the correct ED frame except by using visual inspection.

Table 2 presents the detection results of the ES frame on 10 subjects. The deviation of the ES frame also occurs in two or three consecutive frames, especially in the mid-ventricular slice as the area of the blood pool in the ES frame is smaller than in the ED frame. All these make it more difficult to identify the correct ES frame. The detection accuracy of the ES frame is decreased. Nevertheless, the proposed method could correctly detect the end-expiration in each slice and find the ED and ES frames at the end-expiration in sequence images for the free-breathing CMR imaging as compared with the previous techniques.

Table 3 presents the detection accuracy of our proposed method. The average accuracy of the ED and ES frames of all slices is 76.5%. The average accuracy without the first slice (apex) and the tenth slice (base) is 92.5%. The accuracy is computed as the ratio of the number of correct frames and the total number of frames. The respiratory motion can be depicted according to the cardiac motion. It is difficult to detect the LV center aiming at the respiratory motion fitting. Figure 10 shows images in apical and basal slices. Based on these images, we cannot find the LV chamber. These images would decrease the detection accuracy. We combine the ellipse fitting of the LV area and NCC to find the ED and ES frames at the end-expiration for respiratory motion signal. However, in most studies of LV function assessment, the LV function analysis excludes these slices and the evaluation of the LV function would not be affected. An example is demonstrated in Figure 11 of the segmented LV endocardial contours of the ED frame and ES frame by way of the trained CNN model. As for s1~s9 slices, the segmented contours are on the endocardial border of LV, which can be brought in for detecting the ED and ES frames. As for the tenth slice, the limited number of training subjects leads to inaccurate results. More accurate methods would segment the ED and ES frames with level set [19] or would adopt the endocardial contours from the trained network. Since the aim of this paper is to detect the ED and ES frames, advancing the LV segmentation method will be our further research work.

Finally, one difficulty in detecting the center of LV of the free-breathing CMR data is the smallness of the training dataset, especially for the CNN that requires a large amount of image data. In order to overcome this problem, the STACOM 2011 data is introduced for network training in the first step. Although the STACOM 2011 data have many subjects, the imaging parameters and methods are different between the two distinct datasets in our experiment. The network requires several hours of parameter training. In order to reduce the training process and improve the accuracy of detection of the ED and ES frames, it is necessary to obtain more subjects of the free-breathing CMR data. We will collect more data from volunteers and test them in Deep Learning Network in the near future.

## 6. Conclusion

The presented method requires some manual operation in the training process, but then, the LV center, respiratory motion signal, and the ED and ES frames can be detected automatically in our assembly line. The proposed technique adopted the CNN of deep learning framework to detect LV. The STACOM 2011 database was brought in to train the neural network for the heart region localization, which is an important pretreatment for processing free-breathing CMR data. The LV ellipse fitting method was employed to acquire the respiratory motion signal. The minimum of the normalized cross-correlation and the area of LV ellipse fitting were utilized to identify the ED and ES frames. The results showed that the presented method has high accuracy and stability. Compared with the visual inspection and previous techniques, the improvement for the detection of the ED and ES frames is substantial. This technique greatly reduces the postprocessing time, normally taken by visual identification, and facilitates the application of free-breathing CMR imaging in clinical settings. In the future, we will focus on fully automatic segmentation of the ED and ES frames in free-breathing CMR imaging.

## Figures and Tables

**Table 1:** The detection results of the ED frame in 10 subjects from the first slice (s1) to the tenth slice (s10). ED ± *n* denotes the deviation of the ED detection in n frames. T and F represent the true and false detection results, respectively.

Subject	1	2	3	4	5	6	7	8	9	10
s1	F	F	T	T	F	F	T	F	F	F
s2	T	T	T	T	T	T	T	T	T	T
s3	T	T	T	T	T	T	T	T	T	T
s4	T	T	T	T	T	T	T	T	T	T
s5	T	T	T	T	T	T	T	T	T	T
s6	T	T	T	T	T	T	T	T	T	T
s7	T	T	T	T	T	T	T	T	T	T
s8	T	T	T	T	T	T	T	T	T	ED − 1
s9	T	ED + 1	T	F	T	T	T	T	T	ED + 1
s10	F	F	F	F	F	F	F	F	F	F

**Table 2:** The detection results of the ES frame in 10 subjects from the first slice (s1) to the tenth slice (s10). ES ± *n* denotes the ES detection deviation in n frames. T and F represent the true and false detection results, respectively.

Subject	1	2	3	4	5	6	7	8	9	10
s1	F	F	F	T	F	F	T	F	F	F
s2	T	T	T	ES + 1	F	T	T	T	T	T
s3	T	T	T	T	T	T	T	T	T	T
s4	T	T	T	T	ES − 1	T	T	T	T	T
s5	T	T	T	T	T	T	T	T	T	T
s6	T	T	T	T	T	T	T	T	T	T
s7	T	T	T	T	T	T	T	T	T	T
s8	T	T	T	T	T	T	T	T	T	T
s9	T	T	ES − 1	F	T	ES + 2	T	T	ES + 2	ES + 1
s10	F	F	F	F	F	F	F	F	F	F

**Table 3:** Detection accuracy of the proposed method on 10 subjects.

Subject	1	2	3	4	5	6	7	8	9	10	Average value
Accuracy (including apical and basal slices)	80%	75%	80%	75%	70%	75%	90%	80%	75%	65%	76.5%
Accuracy (without apical and basal slices)	100%	93.75%	93.75%	81.25%	87.5%	93.75%	100%	100%	93.75%	81.25%	92.5%
